# Population characteristics, prescription patterns and glycemic control of users of flash glucose monitoring systems in Brazil: a real-world evidence study

**DOI:** 10.1186/s13098-025-01610-1

**Published:** 2025-02-03

**Authors:** Karla Santo, Josué Nieri, Karine Risério, Karla F. S. Melo

**Affiliations:** 1 Hospital Israelita Albert Einstein, São Paulo, Brazil; 2 Glic™, São Paulo, Brazil; 3 Universidade Federal de São Paulo, São Paulo, Brazil; 4 Sociedade Brasileira de Diabetes, São Paulo, Brazil; 5 Hospital Universitário Lauro Wanderley, Universidade Federal da Paraíba, João Pessoa, Brazil

**Keywords:** Digital health, Real-world evidence, Diabetes, Flash glucose monitoring system, Low-middle income countries

## Abstract

**Background:**

To date, there is a lack of information on the use of flash glucose monitoring system (fCGM) in low-middle income countries, such as Brazil, as well as on digital health platforms most used to calculate the bolus insulin dose. In this study, we aimed to describe the population characteristics, prescription patterns and glycemic control of fCGM users compared to blood glucose monitoring (BGM) system in those who use Glic™, a digital health platform in Brazil, and to assess factors associated with better glycemic control in this population.

**Methods:**

This study is a cross-sectional retrospective study using anonymized aggregated data manually inputted by Glic™ users who self-reported a diagnosis of type 1 diabetes (T1DM), type 2 diabetes (T2DM), gestational diabetes (GDM) and latent autoimmune diabetes in adults (LADA).

**Results:**

Of the 12,727 individuals included in this study, 11,007 (86.5%) reported their glucose monitoring method to be BGM, while 1720 (13.5%) reported using fCGM. Most individuals (70.5%) had T1DM. Compared to BGM, fCGM users were significantly younger, had a higher proportion of males, resided more frequently in the Southeast region of Brazil, had a lower BMI, a longer time since diagnosis, and used Glic™ platform more frequently. fCGM users were prescribed significantly more ultra-long and ultra-rapid acting insulins as their basal and bolus insulin, respectively, and less oral anti-diabetics drugs compared to BGM users. Considering only the T1DM and LADA individuals and their manual glucose inputs, fCGM users had non-significant lower glucose levels than BGM. Use of Glic™ platform and a higher percentage of basal insulin dose were associated with a better glycemic control.

**Conclusion:**

This is the first and largest real-world evidence study that describe and compare fCGM and BGM in users of a digital health patient support platform in Brazil. fCGM users were significantly different from those who perform BGM, in terms of population characteristics and treatment patterns. Glycemic control was better in fCGM users, although not statistically significant due to a restricted sample size. Importantly, a higher frequency of Glic™ use was associated with a higher glucose time in range.

**Supplementary Information:**

The online version contains supplementary material available at 10.1186/s13098-025-01610-1.

## Introduction

The International Diabetes Federation (IDF) has estimated that, globally, there were 537 million adult individuals (aged 20 to 79 years) with diabetes in 2021 [1]. Worryingly, this number is estimated to increase to 783 million by 2045, which represents a 46% increase, and makes diabetes one of the fastest growing medical conditions in the world [1]. Worldwide, approximately 6.7 million adults individuals (aged 20 to 79 years) have died as a result of diabetes and its complications in 2021, corresponding to 12.2% of all global deaths [1]. The economic burden of diabetes on individuals, their families and healthcare systems is significant. The direct costs of diabetes have been estimated to be USD$ 966 billion in 2021, having increase 316% in 15 years [1]. 

In Brazil, in 2022, the prevalence of diabetes was estimated to be approximately 22 million people, and 10 million of those were untreated [2]. In 2018, public spending on diabetes in the Brazilian Unified Health System (SUS) was estimated to be over 1 billion BRL, which accounted for 30% of the total costs related to hypertension, diabetes, and obesity [3]. 

Self-management is a cornerstone of diabetes care. Diabetes self-management is associated with lower HbA1c levels, improved disease knowledge and quality of life, reduced all-cause mortality and reduced healthcare-related costs [4]. An important component of diabetes self-management, which is essential to achieve a good glucose control, is blood glucose monitoring system (BGM) [5]. Although BGM is a proven beneficial strategy in glucose control, adherence to BGM is usually compromised by the need of multiple finger pricks in a day. In the last decade, flash glucose monitoring systems (fCGM), which provides immediate information on interstitial glucose levels, have emerged as a more convenient tool to facilitate glucose monitoring, as this is a small device attached to the skin which prevents diabetic individuals to have to prick their fingers every day [6]. Randomized clinical trials (RCTs) have shown improvements in glycemic control and reductions in hypoglycemia events [7–9]. More recently, real-world evidence (RWE) studies have confirmed benefits, including improvements in glycemic control, treatment satisfaction and well-being, as well as reductions in hypoglycemia, reduced diabetes-related hospitalizations and absenteeism [10–14]. 

In the last few decades, there has been an increasing digitization of healthcare, especially with the larger access to smartphones and the emergence of health apps. In the context of diabetes self-management, health apps are useful tools to personalize the therapy and help patients with their diabetes routine in order to achieve optimal glycemic control. The Glic™ is a digital health support platform freely available (via app and web browser) for people with diabetes and healthcare professionals (physician and dietitians). One of the most used features of Glic™ is the bolus insulin dose calculator, which uses the information about the carbohydrate counting and blood glucose level entered in the platform by the users. The bolus insulin dose calculation is a complex task, as several factors are needed to be considered, including glucose target, carbohydrate-insulin ratio and correction factor for different day periods. The Glic™ platform facilitates this task by automizing these calculations. Additionally, the platform has information on diabetes treatment and has the possibility of setting medication reminders to improve treatment adherence. The users can also share their data with the health professionals who assist them, and the physician and/or dietitian can make prescription changes in the platform guided by the user data patterns. Importantly, the Glic™ platform has had good acceptance by the patients improving the treatment satisfaction by turning the carbohydrates counting into an easier task [15]. 

In this study, we aimed to describe the population characteristics, diabetes treatment patterns and glycemic control of individuals with self-reported diagnosis of type 1 diabetes (T1DM), type 2 diabetes (T2DM), gestational diabetes (GDM) and latent autoimmune diabetes in adults (LADA) who monitor their glucose levels through fCGM compared to BGM, and who use the Glic™ in Brazil. We also aimed to assess factors associated with better glycemic control in this population.

## Methods

### Study design

This study is a cross-sectional retrospective study using anonymized aggregated data available in the Glic™ platform, from 16th September 2021 to 19th October 2023. This study was based on the analysis of the database of a digital health support platform (the Glic™ platform), therefore no research centers were involved in the conduct of this study.

### Study population

The study population consisted of all users of the Glic™ platform, who had self-reported the diagnosis of T1DM, T2DM, GDM or LADA, who had reported the glucose monitoring method (fCGM or BGM) in the platform, and who had recently updated registration information in the last 24 months for adult individuals (aged ≥ 18 years) in the last 6 months for children and adolescents (aged < 18 years) before the date of data extraction.

### Variables of interest and procedures

In this study, the variables of interest included sociodemographic data (age, sex, and geographic region), clinical data (type of diabetes, years of diabetes diagnosis, weight, and height), prescription data (oral antidiabetics, and insulin type, molecule, total daily dose, and number of daily injections), glucose level data (24 h, nocturnal, pre-prandial, and post-prandial) and Glic™ usage data (frequency of use based on glucose level data input).

All data were entered in the Glic™ platform by either the users, their family members or health professionals. Data on glucose levels in the Glic™ platform were manually entered in the app by all users, even for fCGM users, as an automated connection to transmit data from the fCGM device to the Glic™ app was not available. Of note, no laboratory results information, such as those of glycated hemoglobin, are collected in the Glic™ platform. As Glic™ is a patient-oriented platform, routine data quality checks and queries are not performed at the data entry moment. Any data inconsistencies were dealt with in the statistical analyses.

For this study, several data management procedures were implemented to ensure data protection, security, privacy, and confidentiality. These procedures included: (1) separation of the data of the study population from the rest of Glic™ database to create a specific separate dataset with the variables of interest for this study; (2) anonymization of the data, which consisted of removal of all identifiable information from the direct identifiers data fields, such as: full name, telephone number, date of birth and social security number. During the removal of this identifiable information, the user ID in the platform was changed to a new ID with no possibility of returning to the original ID, so there was no possibility of re-identification. Therefore, the data is considered de-identified.

### Statistical analysis

Quantitative variables were summarized as mean and standard deviation. Qualitative variables were summarized in crude frequencies and percentages. Missing data were not counted in percentages.

For quantitative variables, we used the Wilcoxon-Mann-Whitney test to compare two groups and the Kruskal-Wallis test to compare more than two groups. For qualitative variables, we used the Pearson Chi-square test to compare groups.

Some variables were removed or treated in the analyses due data inconsistencies, which could have occurred due to possible errors during the data entry by Glic™ platform users. We removed from the dataset 28 individuals whose data were inputted in the Glic™ platform as GDM in the type of diabetes field and age less than 13 years or male sex, as well as those with data inputted as T2DM and age less than 6 years. We also removed the data on years of diabetes diagnosis for those in which the years of diagnosis were higher than the age. In addition, we removed data on the basal insulin prescription, when basal insulin was inserted as short-acting analogs, ultrashort-acting analogs and human regular. Furthermore, we removed data when the insulin total daily doses were above 200 U/day, as these are not clinically plausible.

Additional data treatment consisted of the following: (a) weight values ≥ 1000 kg were divided by 100 and values ≥ 130 kg and < 1000 kg were divided by 10; (b) height values < 3 m were multiplied by 100 and values ≥ 3 and < 35 were multiplied by 10. After this treatment, for children and adolescents (aged < 18 years), weight and height values were checked against the World Health Organization (WHO) tables of normal range of weight and height for each age. Weight and height outside the WHO normal range were disregarded and not included in the analyses of the z-score. In addition, for adults, weight < 30 kg, and height < 130 cm or > 250 cm were disregarded. All variables that were weight-dependent, such as insulin daily dose per kilogram, were not calculated for those with the weight disregarded. Analyses of the daily bolus insulin dose as well as total daily insulin dose were only calculated for those who had entered information on at least 3 meals per day in the Glic™ platform. The percentage of basal insulin was calculated using the total basal dose in the total daily dose (TBD/TDD). The frequency of Glic™ use was calculated based on the total number of glucose measurements manually inserted in the app per individual during the data extraction time period.

To standardize the analyses of glycemic control to have more robust inferences, we only considered those individuals who had inserted in the Glic™ platform at least one glucose measurement per day in five different days in a week in the last four weeks. In addition, glucose levels < 20 mg/dL were disregarded and excluded from the analyses, as glucometers do not provide values of glucose < 20 mg/dL. The mean glucose level was calculated as a daily weighted average per user for the number of days that there were glucose measurements. Then, the mean of the study population was calculated using this daily weighted average per user. The glucose levels were categorized according to the International Consensus on Time in Range [16], as follows: normoglycemia (glucose levels between 70 mg/dL and 180 mg/dL), hyperglycemia level 1 (glucose levels between 181 mg/dL and 250 mg/dL), hyperglycemia level 2 (glucose levels > 250 mg/dL), hypoglycemia level 1 (glucose levels between 54 mg/dL and 69 mg/dL), and hypoglycemia level 2 (glucose levels < 54 mg/dL). The proportion of each category of glucose level was calculated as the daily weighted proportions per user for the number of days that there were glucose measurements. Then, the proportion of glucose levels of the study population was calculated using this daily weighted average per user.

To investigate factors associated with a better glycemic control, we performed a logistic regression model using the glucose levels (euglycemia vs. dysglycemia) as the dependent variable and the available self-reported sociodemographic, clinical and prescription data as the independent variables. In the logistic regression model, we used the value of the daily weighted average per user to categorize the glycemic control as euglycemia (values categorized as normoglycemia) or dysglycemia (the values categorized as hyperglycemia level 1, hyperglycemia level 2, hypoglycemia level 1 or hypoglycemia level 2). We present the logistic regression results as odds ratios (OR) with their respective Wald 95% confidence interval (CI) and p-values (a two-sided p-value of < 0.05 was considered statistically significant). All analyses and summaries were performed using R software, version 4.1.1 (R Foundation).

An additional analysis was conducted on individuals who switched their glucose monitoring method from BGM to fCGM. For this analysis, we only considered individuals who have registered a change in the glucose monitoring method in the Glic™ platform and who had at least one glucose measurement per day in five different days in a week in the 12 weeks before and after the switch from BGM to fCGM.

### Ethics approval

The study was approved by the Hospital Israelita Albert Einstein Human Research Ethics Committee (CAAE 65669022.0.0000.0071) and a waiver of individual consent was granted for this study.

## Results

### Population characteristics

#### Overall

Of a total of 231,102 Glic™ platform users, 12,727 individuals were eligible based on the study eligibility criteria. Their data were extracted on 19th October 2023 and these individuals were included in this study. Of those included, 11,007 (86.5%) reported their glucose monitoring method to be BGM, while 1720 (13.5%) reported using fCGM. Overall, 70.5% of individuals had T1DM, 20.9% T2DM, 4.5% GDM and 4.1% LADA. There was a significant higher proportion of individuals with T1DM among fCGM users (T1DM 87.6% vs. other types of diabetes 12.4%) compared to BGM (T1DM 67.8% vs. other types of diabetes 32.2%) (*p* < 0.001). The mean overall age was 35.61 years (SD 16.14), and fCGM users were significantly younger (31.62 years, SD 17.32) than those who perform BGM (36.23 years, SD 15.86) (*p* < 0.001). Overall, 39.1% were male, with a significant higher proportion of males among fCGM users (41.9%) compared to BGM (38.6%) (*p* = 0.011). Most individuals (57.0%) were from the Southeast region of Brazil, followed by 17.6% from the South region, 13.7% Northeast region, 9.1% Central-west region and 2.5% North region. There was a significant higher percentage of fCGM users (59.0%) in the Southeast region compared to BGM (56.7%) (*p* < 0.001). The mean number of years of diabetes diagnosis was 9.10 years (SD 10.41), with fCGM users having a significant longer diagnosis time (10.65 years, SD 11.51) compared to BGM (8.87 years, SD 10.21) (*p* < 0.001). The mean body mass index (BMI) was 25.66 kg/m^2^ (SD 5.34), with fCGM users having a significant lower BMI (23.73 kg/m^2^, SD 4.60) compared to BGM (25.94 kg/m^2^, SD 5.38) (*p* < 0.001). Meanwhile, the mean Z-score in children and adolescents (age < 18 years) was 0.13 (SD 1.05), with no significant difference between fCGM and BGM (*p* = 0.408). In terms of use of the Glic™ platform, on average Glic™ users used the platform to input their glucose levels 35.82 times (SD 125.43), with fCGM users using Glic™ more frequently (50.84 times, SD 241.41) than BGM (33.48 times, SD 95.13) (*p* = 0.009). Table 1 presents the characteristics of the study population.

**Table 1:** Characteristics of the study population

	Flash glucose monitoring system *N* = 1720 (13.5%)	Blood glucose monitoring system *N* = 11,007 (86.5%)	Total *N* = 12,727 (100%)	*P* value
**Type of diabetes**				< 0.001
Type 1	1507 (87.6%)	7466 (67.8%)	8973 (70.5%)	
Type 2	85 (4.9%)	2573 (23.4%)	2658 (20.9%)	
Gestational	11 (0.6%)	562 (5.1%)	573 (4.5%)	
LADA	117 (6.8%)	406 (3.7%)	523 (4.1%)	
**Age**				< 0.001
Mean (SD)	31.62 (17.32)	36.23 (15.86)	35.61 (16.14)	
**Age ranges**				< 0.001
< 18 years	376 (21.9%)	742 (6.7%)	1118 (8.8%)	
≥ 18 years	1344 (78.1%)	10,265 (93.3%)	11,609 (91.2%)	
**Sex**				0.011
Female	954 (58.1%)	6539 (61.4%)	7493 (60.9%)	
Male	688 (41.9%)	4115 (38.6%)	4803 (39.1%)	
Missing	78	353	431	
**Region**				< 0.001
Central-west	145 (11.1%)	829 (8.8%)	974 (9.1%)	
Northeast	80 (6.1%)	1387 (14.8%)	1467 (13.7%)	
North	28 (2.1%)	243 (2.6%)	271 (2.5%)	
Southeast	774 (59.0%)	5325 (56.7%)	6099 (57.0%)	
South	284 (21.7%)	1600 (17.1%)	1884 (17.6%)	
Missing	409	1623	2032	
**Years of diabetes diagnosis**				< 0.001
Mean (SD)	10.65 (11.51)	8.87 (10.21)	9.10 (10.41)	
Missing	207	1095	1302	
**BMI (kg/m** ^**2**^ **)**				< 0.001
Mean (SD)	23.73 (4.60)	25.94 (5.38)	25.66 (5.34)	
Missing	203	742	945	
**Z-score** ^*^				0.408
Mean (SD)	0.13 (0.97)	0.13 (1.10)	0.13 (1.05)	
Missing^**^	131	303	434	
**Frequency of Glic™ use**				0.009
Mean (SD)	50.84 (241.41)	33.48 (95.13)	35.82 (125.43)	

BMI, body mass index; LADA, latent autoimmune diabetes in adults; SD, standard deviation

^*^Only for children and adolescents (age < 18 years)

** Considering only children and adolescents (age < 18 years)

#### Comparison of different types of diabetes

Comparing the different types of diabetes, individuals with T1DM were younger (30.85 years, SD 14.48) than those with GDM (33.55 years, SD 5.95), LADA (41.31 years, SD 12.58) and T2DM (51.00 years, SD 13.45). Interestingly, only for T1DM, fCGM users were younger (29.39 years, SD 16.34) than those who perform BGM (31.15 years, SD 14.05) (p = < 0.001). On the contrary, for T2DM, fCGM users were older than those who perform BGM, with mean ages of 55.37 years (SD 15.70) and 50.86 years (SD 13.35) (*p* = 0.002) in T2DM. Meanwhile, for GDM and LADA, there were no significant differences in age between fCGM and BGM. Majority of individuals were female for T1DM (62.4%) and LADA (67.9%); however, most individuals were male for T2DM (53.7%). Similarly to the overall study population, there were more males using fCGM compared to BGM in T1DM (41.5% vs. 36.9%, *p* = 0.001), while there were no statistically significant differences in sex percentages in T2DM and LADA between the two glucose monitoring types. Regarding the country region, for T1DM, GDM and LADA, there were similar regional distribution to the overall study population, with more individuals living in the Southeast region (56.8%, 59.3%, and 60.6%, respectively), followed by South region (18.8%, 17.5%, and 18.5%, respectively), Northeast region (12.7%, 13.4%, and 12.0%, respectively), Central-west region (9.7%, 7.7%, and 7.0%, respectively), and North region (2.0%, 2.1%, and 1.9%, respectively). However, only for T1DM there was statistically significant difference in region distribution comparing those using fCGM compared to BGM, with a higher proportion of fCGM users living in the Southeast region compared to BGM (59.4% vs. 56.3%, *p* < 0.001). For T2DM, there was a different pattern, with more individuals living in the Southeast region (56.7%), followed by Northeast region (17.2%), South region (13.6%), Central-west region (7.9%), and North region (4.6%), but also with no statistically significant difference in region distribution between individuals using fCGM compared to BGM. In terms of years of diabetes diagnosis, T1DM individuals had the longer diagnosis time (11.29 years, SD 10.81), followed by LADA (6.99 years, SD 7.58), T2DM (4.58 years, SD 8.03) and GDM (0.83 years, SD 1.39). For T1DM, fCGM users had a shorter diagnosis time compared to BGM, with mean number of years of diabetes diagnosis of 11.02 (SD 11.72) and 11.35 (SD 10.62) (p = < 0.001), respectively. Conversely, for T2DM and GDM, fCGM users had longer diagnosis time compared to BGM, with mean number of years of diabetes diagnosis of 9.78 (SD 11.88) and 4.41 (SD 7.81) (p = < 0.001) in T2DM, and of 3.00 (SD 5.64) and 0.79 (SD 1.16) (*p* = 0.003) in GDM. Meanwhile, there were no significant differences in years of diabetes diagnosis between LADA individuals who used fCGM or BGM (*p* = 0.444). Individuals with T2DM (29.33 kg/m^2^, SD 5.45) and GDM (29.08 kg/m^2^, SD 5.85) had a higher BMI than LADA (25.12 kg/m^2^, SD 4.26) and T1DM (24.33 kg/m^2^, SD 4.65). Similarly to the overall study population, the BMI was lower in fCGM users compared to BGM in T1DM and T2DM, while there were no statistically significant differences in GDM and LADA. Regarding the frequency of Glic™ use, individuals with LADA (47.11 times, SD 128.17) were the ones who used Glic™ more frequently, followed by T1DM (42.24 times, SD 132.21), GDM (24.35 times, SD 48.11) and T2DM (14.42 times, SD 109.37). Contrary to the overall study population, there were no statistically significant differences in frequency of Glic™ use between fCGM users and BGM in all four types of diabetes. Supplementary Tables 1, 2, 3 and 4 presents the characteristics of the study population by type of diabetes.

### Prescription patterns

#### Overall

In terms of basal insulin prescription, overall, 64.8% had a prescription, with more fCGM users being prescribed a basal insulin (83.8%) than those who perform BGM (61.8%). The type of basal insulin most used were long-acting analogs (62.2%), followed by ultralong-acting analogs (19.3%) and NPH (17.8%). Among the long-acting analogs, Glargine was the most preferred insulin (98.1%), meanwhile among ultralong-acting analogs it was Degludec (93.9%). Comparing basal insulin prescription patterns, fCGM users were prescribed ultralong-acting analogs more frequently (43.5%) than those who perform BGM (14.2%) (*p* < 0.001). Regarding basal insulin doses, the mean total basal daily dose was 26.73 units/day (SD 15.35) and the mean total basal daily dose per kilogram was 0.41 units/kg/day (SD 0.21), with fCGM users having significantly lower doses than BGM, with mean total basal daily doses of 22.14 units/day (SD 13.74) and 27.68 units/day (SD 15.49) (*p* < 0.001), respectively, and the mean total basal daily doses per kilogram of 0.36 units/kg/day (SD 0.19) and 0.42 units/kg/day (SD 0.22) (*p* < 0.001), respectively. The mean number of daily injections of basal insulin was 1.48 (SD 1.42), with fCGM users having significantly lower mean number of daily injections (1.20, SD 0.80) than BGM (1.53, SD 1.51) (*p* < 0.001). The mean percentage of TBD/TDD was 56% (SD 18%), with fCGM having significantly lower ratio (55%, SD 17%) than BGM (57%, SD 18%) (*p* = 0.015).

In terms of bolus insulin prescription, overall, 55.5% had a prescription, with more fCGM users being prescribed bolus insulin (79.2%) than those who perform BGM (51.8%). The type of bolus insulin most used were short-acting analogs (74.7%), followed by ultrashort-acting analogs (16.1%) and human regular (9.2%). Among the short-acting analogs, aspart the most preferred insulin (53%), followed by glulisin (23.9%) and lispro (23.2%). Comparing bolus insulin prescription patterns, fCGM users were prescribed ultrashort-acting analogs more frequently (37.2%) than those who perform BGM (11.0%) (*p* < 0.001). The mean total bolus daily dose was 22.77 (SD 22.61) units/day, with fCGM users having significantly lower doses (19.93, SD 18.61) than BGM (23.45, SD 23.43) (*p* < 0.001). The mean total bolus daily dose per kilogram was 0.36 (0.35) units/kg/day, with no significant differences between fCGM and BGM.

In terms of oral anti-diabetics, only 12.4% of the study population had a prescription of an oral anti-diabetic, with fCGM having a significantly lower percentage of use (6.4%) than BGM (13.3%) (*p* < 0.001). Of the different classes of oral anti-diabetics, metformin was the most used (51.2%), followed by combinations of metformin + sulfonylurea (9.5%), combinations of metformin + SGLT2 inhibitors (9.3%), and SGLT2 inhibitors (6.4%), among other classes and combinations. SGLT2 inhibitors were used in a significantly higher percentage of fCGM users (18.2%) than those who perform BGM (5.5%) (*p* < 0.001). Table 2 presents detailed information on treatment patterns.

**Table 2:** Prescription patterns of the study population

	Flash glucose monitoring system (*N* = 1720)	Blood glucose monitoring system (*N* = 11,007)	Total (*N* = 12,727)	*P* value
**Basal insulin prescription**	1441 (83.8%)	6806 (61.8%)	8247 (64.8%)	< 0.001
**Basal insulin class**				< 0.001
** Long-acting analog**	765 (53.1%)	4366 (64.1%)	5131 (62.2%)	
* Detemir*	18 (2.4%)	81 (1.9%)	99 (1.9%)	
* Glargine*	747 (97.6%)	4285 (98.1%)	5032 (98.1%)	
** Long-acting analog + GLP1**	0 (0%)	1 (0%)	1 (0%)	
* Glargine + Lixisenatida*	0 (0%)	1 (100%)	1 (100%)	
** Ultra-long acting analog**	627 (43.5%)	967 (14.2%)	1594 (19.3%)	
* Degludec*	599 (95.5%)	897 (92.8%)	1496 (93.9%)	
* Glargine U300*	28 (4.5%)	70 (7.2%)	98 (6.1%)	
** Ultra-long acting analog + GLP1**	7 (0.5%)	35 (0.5%)	42 (0.5%)	
* Degludec + Liraglutida*	7 (100%)	35 (100%)	42 (100%)	
** Human NPH**	41 (2.8%)	1427 (21.0%)	1468 (17.8%)	
** Mix**	1 (0.1%)	10 (0.1%)	11 (0.1%)	
* Humalog Mix 25*	1 (100%)	3 (30%)	4 (36.4%)	
* Humalog Mix 50*	0 (0%)	2 (20%)	2 (18.2%)	
* Humulin 70/30*	0 (0%)	4 (40%)	4 (36.4%)	
* NovoMix 30*	0 (0%)	1 (10%)	1 (9.1%)	
Missing	279	4201	4480	
**Basal insulin – Total daily dose (U/day)**				< 0.001
Mean (SD)	22.14 (13.74)	27.68 (15.49)	26.73 (15.35)	
Missing	238	3866	4104	
**Basal insulin – Total daily dose per kilogram (U/Kg/day)**				< 0.001
Mean (SD)	0.36 (0.19)	0.42 (0.22)	0.41 (0.21)	
Missing	344	4111	4455	
**Basal insulin–Number of daily injections**				< 0.001
Mean (SD)	1.20 (0.80)	1.53 (1.51)	1.48 (1.42)	
Missing	236	3858	4094	
**Bolus insulin prescription**	1363 (79.2%)	5699 (51.8%)	7062 (55.5%)	< 0.001
**Bolus insulin class**				< 0.001
** Short-acting analog**	831 (61.0%)	4446 (78.0%)	5277 (74.7%)	
* Aspart*	439 (52.8%)	2357 (53.0%)	2796 (53.0%)	
* Glulisine*	159 (19.1%)	1100 (24.7%)	1259 (23.9%)	
* Lispro*	233 (28.0%)	989 (22.2%)	1222 (23.2%)	
** Ultra-short acting analog**	507 (37.2%)	629 (11.0%)	1136 (16.1%)	
* Fiasp*	507 (100%)	629 (100%)	1136 (100%)	
**Human Regular**	25 (1.8%)	624 (10.9%)	649 (9.2%)	
Missing	357	5308	5665	
**Bolus insulin – Total daily dose (U/day)**				< 0.001
Mean (SD)	19.93 (18.61)	23.45 (23.43)	22.77 (22.61)	
Missing	1068	8323	9391	
**Bolus insulin – Total daily dose per kilogram (U/Kg/day)**				0.566
Mean (SD)	0.34 (0.30)	0.36 (0.37)	0.36 (0.35)	
Missing	1123	8413	9536	
**Bolus insulin – Correction factor 24 h**				< 0.001
Mean (SD)	52.77 (30.00)	47.92 (59.52)	48.83 (55.23)	
Missing	1124	8428	9552	
**Bolus insulin – Carbohydrate/insulin ratio**				0.076
Mean (SD)	13.91 (9.60)	14.53 (21.53)	14.41 (19.84)	
Missing	1123	8424	9547	
**TBD/TDD (%)**				0.015
Mean (SD)	55 (17)	57 (18)	56 (18)	
Missing	1094	8417	9511	
**Oral anti-diabetics prescription**	110 (6.4%)	1464 (13.3%)	1574 (12.4%)	< 0.001
**Oral anti-diabetics class**				< 0.001
* Metformin*	46 (41.8%)	760 (51.9%)	806 (51.2%)	
* Sulfonylureas*	0 (0.0%)	62 (4.2%)	62 (3.9%)	
* Glitazones*	1 (0.9%)	7 (0.5%)	8 (0.5%)	
* DPP4 inhibitors*	7 (6.4%)	26 (1.8%)	33 (2.1%)	
* GLP1 agonists*	4 (3.6%)	19 (1.3%)	23 (1.5%)	
* SGLT2 inhibitors*	20 (18.2%)	81 (5.5%)	101 (6.4%)	
* Acarbose*	1 (0.9%)	6 (0.4%)	7 (0.4%)	
* Metformin + Sulfonylurea*	0 (0.0%)	149 (10.2%)	149 (9.5%)	
* Metformin + SGLT2 inhibitors*	15 (13.6%)	131 (8.9%)	146 (9.3%)	
* Metformin + DPP4 inhibitors*	5 (4.5%)	51 (3.5%)	56 (3.6%)	
* Other combination drugs*	11 (10.0%)	172 (11.7%)	183 (11.6%)	

DPP4, dipeptidyl peptidase 4; GLP1, glucagon-like peptide 1; SD, standard deviation; SGLT2, sodium-glucose transport protein 2; TBD/TDD, total basal dose and total daily dose of insulin; U, units

#### Comparison of different types of diabetes

Comparing the different types of diabetes, individuals with LADA had the highest percentage of basal insulin prescription (88.5%), followed by T1DM (79.5%), T2DM (22.8%) and GDM (7.3%), with more fCGM users being prescribed basal insulin than those who perform BGM, except for LADA for which there were not significant difference. The type of basal insulin most used were long-acting analogs for T1DM (65.8%) and LADA (55.7%), while human NPH was the one most used for GDM (83.3%) and T2DM (58.7%). Comparing basal insulin prescription patterns, fCGM users were prescribed ultralong-acting analogs more frequently than those who perform BGM for all types of diabetes. Regarding bolus insulin treatment, individuals with LADA had the highest percentage of bolus insulin prescription (72.1%), followed by T1DM (71.3%), T2DM (10.1%) and GDM (2.6%), with more fCGM users being prescribed bolus insulin than those who perform BGM in all types of diabetes, except LADA. The type of bolus insulin most used were short-acting analogs for T1DM (77.0%) and LADA (66.3%), while human regular was the one most used for GDM (60.0%) and T2DM (45.4%). Comparing bolus insulin prescription patterns, fCGM users were prescribed ultrashort-acting analogs more frequently than those who perform BGM for all types of diabetes, except GDM. More detailed information on the prescription patterns per type of diabetes are presented in Supplementary Tables 5, 6, 7 and 8.

### Glycemic control

In terms of glucose data manually inserted in the app by the user, there were only 841 individuals in the study population of Glic™ app users who attended the criteria for the glycemic control analyses. Of those, 657 had T1DM, 96 had T2DM, 47 had GDM and 41 had LADA. Considering that T1DM and LADA represented more than 80% (698/841) of this glucose data cohort, have similarities in terms of pathophysiology of endogenous insulin deficiency and treatment, and that the population profile of these two types of diabetes was similar between fCGM users and BGM (See Supplementary Table 9), the glucose data analyses were grouped for these two types of diabetes. Comparisons of population profile between all eligible patients and this glucose data cohort are also presented in Supplementary Table 10. The mean 24 h glucose level was 180.24 mg/dL (SD 46.76), with fCGM users having a non-significant lower glucose level (174.58 mg/dL, SD 39.40) than those who perform BGM (181.41 mg/dL, SD 48.10) (*p* = 0.212). When this population of T1DM and LADA were split into adults (> 18 years) and children and adolescents (< 18 years), the mean 24 h glucose level in adults was lower (179.04 mg/dL, SD 47.34) than in children and adolescents (185.69 mg/dL, SD 43.81). In both age groups, fCGM users had a non-significant lower glucose level than those who perform BGM, with glucose levels of 174.27 mg/dL (SD 38.90) and 179.80 mg/dL (SD 48.55) (*p* = 0.467) in adults and of 175.18 mg/dL (SD 40.83) and 190.75 mg/dL (SD 44.52) (*p* = 0.072) in children and adolescents, respectively. Table 3 presents the detailed glucose levels analyses for the T1DM and LADA individuals, including nocturnal, pre-prandial and post-prandial glucose levels. Supplementary Tables 11 and 12 presents the detailed glucose levels analyses for the adults and children and adolescents with T1DM and LADA. For T2DM, the mean 24 h, nocturnal, pre-prandial and post-prandial glucose levels were 144.28 mg/dL (SD 48.33), 156.36 mg/dL (SD 70.19), 139.77 mg/dL (SD 39.95), 161.98 mg/dL (SD 68.53), respectively. For GDM, the mean 24 h, nocturnal, pre-prandial and post-prandial glucose levels were 102.47 mg/dL (SD 9.02), 110.24 mg/dL (SD 17.95), 88.43 mg/dL (SD 7.76), 112.12 mg/dL (SD 9.65), respectively. Comparisons between fCGM users and BGM were not possible for these two types of diabetes as there were only two and one fCGM users among these T2DM and GDM individuals, respectively.

**Table 3:** Glucose levels of the type 1 diabetes and LADA individuals who attended the glycemic control analyses requirements

	Flash glucose monitoring system (*N* = 120)	Blood glucose monitoring system (*N* = 578)	Total (*N* = 698)	*P* value
**24 h glucose level (mg/dL)**				0.212
Mean (SD)	174.58 (39.40)	181.41 (48.10)	180.24 (46.76)	
Missing	0	0	0	
**Nocturnal glucose level (mg/dL)**				0.007
Mean (SD)	226.00 (75.11)	192.38 (86.78)	196.84 (85.97)	
Missing	79	310	389	
**Pre-prandial glucose level (mg/dL)**				0.061
Mean (SD)	167.34 (35.37)	177.07 (47.07)	175.38 (45.38)	
Missing	2	17	19	
**Post-prandial glucose level (mg/dL)**				0.645
Mean (SD)	183.44 (58.62)	187.07 (64.67)	186.43 (63.62)	
Missing	5	42	47	
Glycemic control categories*				
* Normoglycemia*	55%	51%	52%	
* Level 2 hypoglycemia*	1%	2%	1%	
* Level 1 hypoglycemia*	3%	4%	4%	
* Level 1 hyperglycemia*	23%	23%	23%	
* Level 2 hyperglycemia*	17%	21%	20%	

SD, standard deviation

* N not presented as the proportion of glycemic control categories was calculated using the daily weighted average per user

**Fig. 1:**
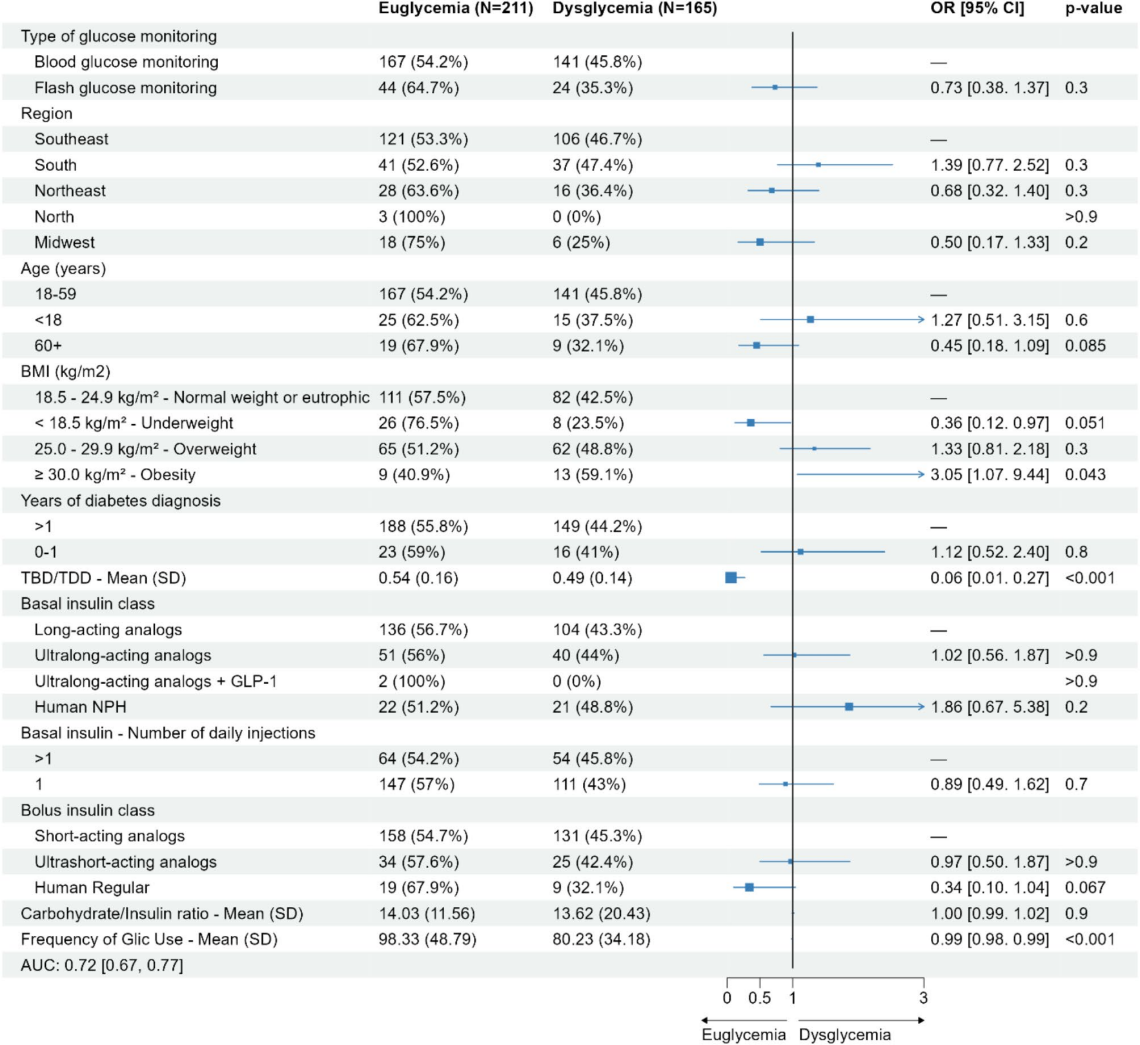
Forest plot of factors associated with a better glycemic control BMI, body mass index, CI, confidence interval; GLP1, glucagon-like peptide 1; OR, odds ratio; SD, standard deviation; TBD/TDD, total basal dose/total daily dose

In terms of factors associated with a better glycemic control, in a population of 376 individuals with all four types of diabetes for which there were complete data, it was found in this present study that a higher frequency of use of Glic™ and a higher percentage of TBD/TDD were associated with a higher odds of being euglycemic. On the contrary, obesity was associated with dysglycemia. The use of fCGM tended to be associated with euglycemia compared to BGM; however, this association was not statistically significant. There were no associations with a better glycemic control for all other factors, including age, country region, years of diabetes diagnosis, class of basal and bolus insulin, number of basal insulin injections and carbohydrate/insulin ratio (Fig. 1).

Regarding the switch of glucose monitoring method from BGM to fCGM, of the 211 individuals included in this analysis, 35% were dysglycemic when using BGM. This number dropped to 30% after switching to fCGM, but without statistical significance (*p* = 0.24). There was also no difference in the therapeutic regimen (basal and bolus insulin) after changing from BGM to fCGM.

## Discussion

This study is the first and largest RWE study to describe and compare the population profile, treatment patterns and glycemic control of fCGM and BGM in a population of users of a real-world digital health patient support platform (Glic™) who have T1DM, T2DM, GDM and LADA in Brazil. We found that, of a total of 12,727 individuals who use the Glic™ platform for diabetes management support, the majority still perform BGM as their glucose monitoring method, with less than 15% of them using fCGM. In addition, most Glic™ users had T1DM and a higher proportion of fCGM users had T1DM in relation to the other types of diabetes when compared to BGM. Importantly, population characteristics of fCGM users were significantly different from those who perform BGM, being younger, having a higher proportion of males, being more concentrated in the Southeast region of Brazil, having a lower BMI, a longer diabetes diagnosis time, and using Glic™ platform more frequently.

The higher proportion of BGM demonstrates that, in a low-middle-income country like Brazil, BGM is still the standard method of monitoring glucose levels, especially because people with insulin-dependent diabetes are provided with glucose monitors and test strips for free by the Brazilian Public Unified Health System, under a national regulation published in 2007 [17, 18]. Also, in Brazil, fCGM was the only standalone continuous glucose monitoring device available until July 2024. In addition, the finding that, in the Glic™ platform and among fCGM users, there were more individuals with T1DM is explained by the fact that people with T1DM usually have to monitor their glucose levels more frequently than other types of diabetes and, thus, the use of fCGM is more convenient [8]. This scenario is confirmed by our study finding that fCGM users use Glic™ more frequently to register their measured glucose levels in the app and have guidance in bolus insulin calculation through the Glic™ carbohydrate counting calculator. Digital health platforms, together with glucose monitoring, enhance blood glucose management by providing real-time data and insulin dosing guidance, offering particular benefits for T1DM individuals [19]. 

Overall, the younger age of fCGM users can be attributed to a higher proportion of T1DM in the study population, as T1DM patients tend to be diagnosed earlier in life. Looking at the four different types of diabetes evaluated in our study, for T1DM, fCGM users were younger than those who perform BGM, while for T2DM, fCGM users were older, and there were no differences for GDM and LADA. In addition, for T1DM, there was a higher percentage of male individuals and those residing in the Southeast region of Brazil in fCGM users compared to BGM; while there were no differences in the other types of diabetes. This finding might be explained by the fact that the Southeast region is the largest populous region of Brazil and is responsible for more than half of Brazil’s gross domestic product (GDP), with higher average incomes, particularly among men, and thus, newer technologies, such as fCGM and apps are usually more readily available for the male population in this region. In terms of years of diabetes diagnosis, for T2DM and GDM, fCGM users had longer diagnosis time compared to BGM, while for T1DM, fCGM users had a slightly shorter diagnosis time, and there was no significant difference in LADA individuals. The lower average age at diagnosis of T1DM compared to T2DM may help explain this, as younger individuals with T1DM might be more inclined to be early adopters [20, 21]. However, further studies regarding association between longer diabetes duration and increased use of advanced technologies are needed. This highlights the multifaceted nature of factors influencing both adherence and the adoption of innovations [22, 23]. Furthermore, the lower BMI observed in fCGM users aligns with findings that individuals with better treatment adherence, including healthier diets and regular physical activity, are more likely to utilize advanced diabetes technologies [24, 25]. These findings suggest that fCGM is appropriate for use and has a high usability irrespective of age, sex and time of diabetes diagnosis.

Regarding prescription patterns, overall fCGM users had a significantly higher percentage of basal and bolus insulin prescription, as well as were prescribed significantly more ultra-long and ultra-short acting analogs as their basal and bolus insulin, respectively, and less oral anti-diabetics compared to BGM. Concerning the different types of diabetes, for those with T1DM, T2DM and GDM, fCGM users were prescribed basal and bolus insulin more often than those who perform BGM, while for LADA there was not a significant difference. Interestingly, the type of basal and bolus insulin most used in T1DM and LADA were long and short-acting analogs, respectively, while NPH and regular insulin were the ones most used for GDM and T2DM, respectively. The higher prescription of insulin analogs in T1DM aligns with Brazilian Diabetes Society guidelines, recommending long-acting and short-acting analogs for better glycemic control and reduced hypoglycemia events [26]. In addition, fCGM users were prescribed ultralong and ultrashort-acting analogs more frequently than those who perform BGM for all types of diabetes, except GDM. The increased use of the Glic™ platform by fCGM users for bolus insulin calculation supports this, as it facilitates insulin dosing accuracy and a better understanding of insulin action on glucose levels. This, in turn, helps select analogs insulin optimizing treatment outcomes.

In our study, we also found that the percentage of basal insulin was significantly lower among fCGM users compared to BGM in those with T1DM. This finding suggests that fCGM may facilitate the identification of hypoglycemic events, which may contribute to subsequent reductions in basal insulin dosage, which may, in turn, reduce further hypoglycemic events. These patterns highlight the role of fCGM in improving insulin therapy [7, 27]. We anticipate that this therapeutic evolution, combined with the incorporation of rapid and long-acting insulin analogs and the availability of digital tools that automate bolus insulin calculations, like Glic™, will lead to improved glycemic control and reduced hypoglycemia events in insulin users in Brazil.

In our study, considering only the T1DM and LADA individuals, fCGM users tended to have lower glucose levels than BGM. Our results were in line with previous evidence from RCTs and RWE studies that showed that fCGM is associated with a better glycemic control [7–11]. However, although fCGM users tended to have a better glucose management in our study, the difference was not statistically significant probably due to the small sample size of the population for which the glucose levels were evaluated. As there were no requirements of minimal frequency of use of the Glic™ platform, we decided to only analyze glucose levels for those individuals who had inserted in the Glic™ platform at least one glucose measurement per day in five different days in a week in the last four weeks to have a more robust inference on the impact of fCGM in the glycemic control. This low number of individuals who inserted a minimum number of glucose measurements might also be influenced by the fact that, even for fCGM users, the glucose level had to be manually inserted in the Glic™ app, as there was no integration between the fCGM device and Glic™ app to do an automatic data transfer. Although, with this strategy, we could guarantee more robust evidence, there was a trade on the number of individuals with the necessary data for the analyses, which, in turn, resulted in less power to detect a difference between groups. Of note, we could identify a few factors that were associated with a better glycemic control. One of these factors is the use of the Glic™ platform in a higher frequency, which is also in accordance with other studies supporting the use of digital health platforms/apps as a strategy to improve glycemic control [28]. 

Our study has a unique strength as it is a RWE study conducted through a digital health patient support platform, which is currently being used by thousands of people with diabetes in a low-middle-income country. Digital health platforms enable us to generate evidence using real-world data in a more efficient way, through the analysis of relevant data on very large populations. However, our study also has some limitations. First, our study evaluated self-reported data and there was no confirmation of diagnosis of diabetes through laboratory results or medical notes in the platform. Second, as the platform is a user-directed platform, no data checks or validation through data queries are performed in the platform, so incorrect data entered in the platform was not verified at the data entry point. We tried to mitigate this issue in the data analysis, in which we did several data treatments as described in the methods. Third, the results presented in this study reflect the findings among individuals with T1DM, T2DM, GDM and LADA who use the Glic™ platform and, therefore, do not represent the whole population of people with diabetes in Brazil. Nonetheless, the insights provided by these results on this very large population of people with diabetes are valuable.

## Conclusion

This is the first and largest RWE study to describe and compare fCGM and BGM, in terms of population characteristics and prescription patterns, in a population of users of a real-world digital health patient support platform (Glic™) with T1DM, T2DM, GDM and LADA in Brazil. In this study, we found that the majority of individuals who use the Glic™ platform for diabetes management support had T1DM and still perform BGM as their glucose monitoring method. Among fCGM users, there was a higher proportion of T1DM individuals as they tend to be earlier adopters of newer technologies such as Glic™ and fCGM sensors given their need to monitor their glucose levels more closely than other types of diabetes. We were able to identify differences in population characteristics and prescription patterns among those using BGM compared to fCGM as well as among the different types of diabetes and these suggest that fCGM is appropriate for use and has a high usability irrespective of age, sex and time of diabetes diagnosis. We also found differences in prescription patterns that corroborates with the fact that the use of the digital health patient support platforms, such as Glic™, together with glucose monitoring via fCGM facilitates insulin dosing accuracy and selection of insulin analogs to optimize treatment outcomes. Finally, glycemic control was found to be better in fCGM users, although not statistically significant due to a restricted sample size. Importantly, a higher frequency of Glic™ use and higher percentage of basal insulin were associated with a better glycemic control.

## Electronic supplementary material

Below is the link to the electronic supplementary material.

Supplementary Material 1

## Data Availability

No datasets were generated or analysed during the current study.

## References

[1] IDF Diabetes Atlas. Brussels, Belgium: International Diabetes Federation; 2021.

[2] Zhou B, Rayner AW, Gregg EW, Sheffer KE, Carrillo-Larco RM, Bennett JE, et al. Worldwide trends in diabetes prevalence and treatment from 1990 to 2022: a pooled analysis of 1108 population-representative studies with 141 million participants. Lancet. 2024;404(10467):2077–93. 39549716 10.1016/S0140-6736(24)02317-1 PMC7616842

[3] Nilson EAF, Andrade R, de Brito DA, de Oliveira ML. [Costs attributable to obesity, hypertension, and diabetes in the Unified Health System, Brazil, 2018Costos atribuibles a la obesidad, la hipertensión Y La Diabetes en El Sistema Único De Salud De Brasil, 2018]. Rev Panam Salud Publica. 2020;44:e32. 32284708 10.26633/RPSP.2020.32 PMC7147115

[4] ElSayed NA, Aleppo G, Aroda VR, Bannuru RR, Brown FM, Bruemmer D, et al. 5. Facilitating Positive Health Behaviors and Well-being to Improve Health outcomes: standards of Care in Diabetes—2023. Diabetes Care. 2022;46(Supplement1):S68–96. 10.2337/dc23-S005 PMC9810478 36507648

[5] Benjamin EM. Self-monitoring of blood glucose: the basics. Clin Diabetes. 2002;20(1):45–7.

[6] Thomas Simon James C, Pratik C, Partha K, Emma GW. Flash glucose monitoring: the story so far and the journey ahead. BMJ Innovations. 2023;9(1):27.

[7] Bolinder J, Antuna R, Geelhoed-Duijvestijn P, Kröger J, Weitgasser R. Novel glucose-sensing technology and hypoglycaemia in type 1 diabetes: a multicentre, non-masked, randomised controlled trial. Lancet. 2016;388(10057):2254–63. 27634581 10.1016/S0140-6736(16)31535-5

[8] Haak T, Hanaire H, Ajjan R, Hermanns N, Riveline JP, Rayman G. Flash glucose-sensing technology as a replacement for blood glucose monitoring for the management of insulin-treated type 2 diabetes: a Multicenter, open-label Randomized Controlled Trial. Diabetes Therapy: Res Treat Educ Diabetes Relat Disorders. 2017;8(1):55–73. 10.1007/s13300-016-0223-6 PMC5306122 28000140

[9] Wada E, Onoue T, Kobayashi T, Handa T, Hayase A, Ito M et al. Flash glucose monitoring helps achieve better glycemic control than conventional self-monitoring of blood glucose in non-insulin-treated type 2 diabetes: a randomized controlled trial. BMJ Open Diabetes Res Care. 2020;8(1). 10.1136/bmjdrc-2019-001115 PMC7292039 32518063

[10] Bailey CJ, Gavin JR 3. Flash continuous glucose monitoring: a Summary Review of recent real-world evidence. Clin Diabetes. 2021;39(1):64–71. 33551555 10.2337/cd20-0076 PMC7839606

[11] Evans M, Welsh Z, Seibold A. Reductions in HbA1c with flash glucose monitoring are sustained for up to 24 months: a Meta-analysis of 75 real-world Observational studies. Diabetes Therapy. 2022;13(6):1175–85. 35476279 10.1007/s13300-022-01253-9 PMC9174370

[12] Roussel R, Riveline JP, Vicaut E, de Pouvourville G, Detournay B, Emery C, et al. Important Drop in Rate of Acute Diabetes complications in people with type 1 or type 2 diabetes after initiation of Flash glucose monitoring in France: the RELIEF study. Diabetes Care. 2021;44(6):1368–76. 33879536 10.2337/dc20-1690 PMC8247513

[13] Miller E, Brandner L, Wright EJ. 84-LB: HbA1c reduction after initiation of the FreeStyle Libre System in type 2 diabetes patients on long-acting insulin or noninsulin therapy. Diabetes. 2020;69(Supplement1):84–LB.

[14] Jaques-Albuquerque LT, Dos Anjos-Martins E, Torres-Nunes L, Valério-Penha AG, Coelho-Oliveira AC, da Silva Sarandy VL et al. Effectiveness of Using the FreeStyle Libre(^®^) System for Monitoring Blood Glucose during the COVID-19 Pandemic in Diabetic Individuals: Systematic Review. Diagnostics (Basel). 2023;13(8). 10.3390/diagnostics13081499 PMC10137815 37189600

[15] Magalhães VAQ, Nery MS, Alves M, Silva C, Fagundes RPS, Melo FDC. K.F.S. Real time support to insulin dose adjustment improves glycemic control. International Congress of Endocrinology; Nov. 8-12th; Rio de Janeiro, Brasil. 2008. Available from: https://gliconline.net/real-time-support-to-insulin-dose-adjustment-improves-glycemic-control/

[16] Battelino T, Danne T, Bergenstal RM, Amiel SA, Beck R, Biester T, et al. Clinical targets for continuous glucose Monitoring Data Interpretation: recommendations from the International Consensus on Time in Range. Diabetes Care. 2019;42(8):1593–603. 31177185 10.2337/dci19-0028 PMC6973648

[17] Coutinho WF, Silva Júnior WS. Diabetes Care in Brazil. Ann Glob Health. 2015;81(6):735–41. 27108141 10.1016/j.aogh.2015.12.010

[18] PORTARIA Nº 2.583, DE, 10 DE OUTUBRO. DE 2007 2007 [Available from: https://bvsms.saude.gov.br/bvs/saudelegis/gm/2007/prt2583_10_10_2007.html

[19] Cahn A, Akirov A, Raz I. Digital health technology and diabetes management. J Diabetes. 2018;10(1):10–7. 28872765 10.1111/1753-0407.12606

[20] Gregory GA, Robinson TIG, Linklater SE, Wang F, Colagiuri S, de Beaufort C, et al. Global incidence, prevalence, and mortality of type 1 diabetes in 2021 with projection to 2040: a modelling study. Lancet Diabetes Endocrinol. 2022;10(10):741–60. 36113507 10.1016/S2213-8587(22)00218-2

[21] Peer N, Balakrishna Y, Durao S. Screening for type 2 diabetes mellitus. Cochrane Database Syst Rev. 2020;5(5):Cd005266. 32470201 10.1002/14651858.CD005266.pub2 PMC7259754

[22] Sandy JL, Tittel SR, Rompicherla S, Karges B, James S, Rioles N, et al. Demographic, clinical, management, and outcome characteristics of 8,004 Young Children with type 1 diabetes. Diabetes Care. 2024;47(4):660–7. 38305782 10.2337/dc23-1317

[23] Sousa C, Neves JS, Dias CC, Sampaio R. Adherence to glucose monitoring with intermittently scanned continuous glucose monitoring in patients with type 1 diabetes. Endocrine. 2023;79(3):477–83. 36574148 10.1007/s12020-022-03288-1 PMC9988994

[24] Marigliano M, Eckert AJ, Guness PK, Herbst A, Smart CE, Witsch M, et al. Association of the use of diabetes technology with HbA1c and BMI-SDS in an international cohort of children and adolescents with type 1 diabetes: the SWEET project experience. Pediatr Diabetes. 2021;22(8):1120–8. 34716736 10.1111/pedi.13274

[25] Lanzinger S, Best F, Bergmann T, Laimer M, Lipovsky B, Danne T, et al. Dynamics of Hemoglobin A1c, body Mass Index, and rates of severe hypoglycemia in 4434 adults with type 1 or type 2 diabetes after initiation of continuous glucose monitoring. Diabetes Technol Ther. 2022;24(10):763–9. 35653726 10.1089/dia.2022.0063

[26] Brazilian Diabetes Society Guidelines. 2024 [Available from: https://diretriz.diabetes.org.br/

[27] Gomes MB, Negrato CA. Adherence to insulin therapeutic regimens in patients with type 1 diabetes. A nationwide survey in Brazil. Diabetes Res Clin Pract. 2016;120:47–55. 27513598 10.1016/j.diabres.2016.07.011

[28] Debong F, Mayer H, Kober J. Real-world assessments of mySugr Mobile Health App. Diabetes Technol Ther. 2019;21(S2):S2–35. 10.1089/dia.2019.0019 31169427

