# Supplementary material for: Population characteristics, prescription patterns and glycemic control of users of flash glucose monitoring systems in Brazil: a real-world evidence study
Source: Diabetol Metab Syndr. 2025 Feb 3;17:44. doi: 10.1186/s13098-025-01610-1 (PMC11789284; doi:10.1186/s13098-025-01610-1)
Supplement: Supplementary file 1 — Supplementary Material 1 [file 13098_2025_1610_MOESM1_ESM.docx]

**Supplementary material**

| **Supplementary Table 1. Type 1 diabetes population characteristics** | | | | |
| --- | --- | --- | --- | --- |
|  | **Flash glucose monitoring system**  **N=1507 (16.8%)** | **Blood glucose monitoring system**  **N=7466 (83.2%)** | **Total**  **N=8973 (100%)** | **P value** |
| **Age** |  |  |  | < 0.001 |
| Mean (SD) | 29.39 (16.34) | 31.15 (14.05) | 30.85 (14.48) |  |
| **Sex** |  |  |  | 0.001 |
| Female | 840 (58.5%) | 4530 (63.1%) | 5370 (62.4%) |  |
| Male | 595 (41.5%) | 2644 (36.9%) | 3239 (37.6%) |  |
| Missing | 72 | 292 | 364 |  |
| **Region** |  |  |  | < 0.001 |
| Central-west | 126 (11.0%) | 594 (9.5%) | 720 (9.7%) |  |
| Northeast | 70 (6.1%) | 874 (13.9%) | 944 (12.7%) |  |
| North | 22 (1.9%) | 123 (2.0%) | 145 (2.0%) |  |
| Southeast | 678 (59.4%) | 3532 (56.3%) | 4210 (56.8%) |  |
| South | 246 (21.5%) | 1150 (18.3%) | 1396 (18.8%) |  |
| Missing | 365 | 1193 | 1558 |  |
| **Years of diabetes diagnosis** |  |  |  | 0.002 |
| Mean (SD) | 11.02 (11.72) | 11.35 (10.62) | 11.29 (10.81) |  |
| Missing | 195 | 932 | 1127 |  |
| **BMI (kg/m^2^)** |  |  |  | < 0.001 |
| Mean (SD) | 23.40 (4.56) | 24.50 (4.64) | 24.33 (4.65) |  |
| Missing | 197 | 580 | 777 |  |
| **Z-score^*^** |  |  |  | 0.391 |
| Mean (SD) | 0.12 (0.97) | 0.13 (1.10) | 0.13 (1.05) |  |
| Missing | 1263 | 7027 | 8290 |  |
| **Frequency of Glic use** |  |  |  | 0.081 |
| Mean (SD) | 51.31 (249.35) | 40.41 (91.89) | 42.24 (132.21) |  |

| **Supplementary Table 2. Type 2 diabetes population characteristics** | | | | |
| --- | --- | --- | --- | --- |
|  | **Flash glucose monitoring system**  **N=85 (3.2%)** | **Blood glucose monitoring system**  **N=2573 (96.8%)** | **Total**  **N=2658 (100%)** | **P value** |
| **Age** |  |  |  | 0.002 |
| Mean (SD) | 55.37 (15.70) | 50.86 (13.35) | 51.00 (13.45) |  |
| **Sex** |  |  |  | 0.098 |
| Female | 31 (37.3%) | 1176 (46.6%) | 1207 (46.3%) |  |
| Male | 52 (62.7%) | 1350 (53.4%) | 1402 (53.7%) |  |
| Missing | 2 | 47 | 49 |  |
| **Region** |  |  |  | 0.066 |
| Central-west | 10 (14.5%) | 174 (7.7%) | 184 (7.9%) |  |
| Northeast | 5 (7.2%) | 397 (17.5%) | 402 (17.2%) |  |
| North | 3 (4.3%) | 104 (4.6%) | 107 (4.6%) |  |
| Southeast | 39 (56.5%) | 1283 (56.7%) | 1322 (56.7%) |  |
| South | 12 (17.4%) | 306 (13.5%) | 318 (13.6%) |  |
| Missing | 16 | 309 | 325 |  |
| **Years of diabetes diagnosis** |  |  |  | < 0.001 |
| Mean (SD) | 9.78 (11.88) | 4.41 (7.81) | 4.58 (8.03) |  |
| Missing | 4 | 118 | 122 |  |
| **BMI (kg/m^2^)** |  |  |  | < 0.001 |
| Mean (SD) | 27.22 (4.44) | 29.40 (5.47) | 29.33 (5.45) |  |
| Missing | 4 | 138 | 142 |  |
| **Frequency of Glic use** |  |  |  | 0.317 |
| Mean (SD) | 17.72 (39.50) | 14.31 (110.93) | 14.42 (109.37) |  |

| **Supplementary Table 3. Gestational diabetes population characteristics** | | | | |
| --- | --- | --- | --- | --- |
|  | **Flash glucose monitoring system**  **N=11 (1.9%)** | **Blood glucose monitoring system**  **N=562 (98.1%)** | **Total**  **N=573 (100%)** | **P value** |
| **Age** |  |  |  | 0.122 |
| Mean (SD) | 36.00 (5.37) | 33.51 (5.95) | 33.55 (5.95) |  |
| **Sex** |  |  |  | NA |
| Female | 11 (100.0%) | 562 (100.0%) | 573 (100.0%) |  |
| Male | 0 (0.0%) | 0 (0.0%) | 0 (0.0%) |  |
| Missing | 0 | 0 | 0 |  |
| **Region** |  |  |  | 0.370 |
| Central-west | 0 (0.0%) | 40 (7.8%) | 40 (7.7%) |  |
| Northeast | 0 (0.0%) | 70 (13.6%) | 70 (13.4%) |  |
| North | 0 (0.0%) | 11 (2.1%) | 11 (2.1%) |  |
| Southeast | 4 (57.1%) | 305 (59.3%) | 309 (59.3%) |  |
| South | 3 (42.9%) | 88 (17.1%) | 91 (17.5%) |  |
| Missing | 4 | 48 | 52 |  |
| **Years of diabetes diagnosis** |  |  |  | 0.003 |
| Mean (SD) | 3.00 (5.64) | 0.79 (1.16) | 0.83 (1.39) |  |
| Missing | 1 | 6 | 7 |  |
| **BMI (kg/m^2^)** |  |  |  | 0.109 |
| Mean (SD) | 26.79 (7.42) | 29.12 (5.82) | 29.08 (5.85) |  |
| Missing | 0 | 16 | 16 |  |
| **Frequency of Glic use** |  |  |  | 0.637 |
| Mean (SD) | 121.73 (257.33) | 22.44 (31.47) | 24.35 (48.11) |  |

| **Supplementary Table 4. LADA population characteristics** | | | | |
| --- | --- | --- | --- | --- |
|  | **Flash glucose monitoring system**  **N=117 (22.4%)** | **Blood glucose monitoring system**  **N=406 (77.6%)** | **Total**  **N=523 (100%)** | **P value** |
| **Age** |  |  |  | 0.113 |
| Mean (SD) | 42.70 (14.01) | 40.91 (12.13) | 41.31 (12.58) |  |
| **Sex** |  |  |  | 0.277 |
| Female | 72 (63.7%) | 271 (69.1%) | 343 (67.9%) |  |
| Male | 41 (36.3%) | 121 (30.9%) | 162 (32.1%) |  |
| Missing | 4 | 14 | 18 |  |
| **Region** |  |  |  | 0.053 |
| Central-west | 9 (9.7%) | 21 (6.3%) | 30 (7.0%) |  |
| Northeast | 5 (5.4%) | 46 (13.8%) | 51 (12.0%) |  |
| North | 3 (3.2%) | 5 (1.5%) | 8 (1.9%) |  |
| Southeast | 53 (57.0%) | 205 (61.6%) | 258 (60.6%) |  |
| South | 23 (24.7%) | 56 (16.8%) | 79 (18.5%) |  |
| Missing | 24 | 73 | 97 |  |
| **Years of diabetes diagnosis** |  |  |  | 0.444 |
| Mean (SD) | 7.57 (7.99) | 6.81 (7.46) | 6.99 (7.58) |  |
| Missing | 7 | 39 | 46 |  |
| **BMI (kg/m^2^)** |  |  |  | 0.292 |
| Mean (SD) | 24.66 (3.52) | 25.26 (4.44) | 25.12 (4.26) |  |
| Missing | 2 | 8 | 10 |  |
| **Frequency of Glic use** |  |  |  | 0.091 |
| Mean (SD) | 62.17 (219.39) | 42.76 (85.46) | 47.11 (128.17) |  |

| **Supplementary Table 5 – Prescription patterns in type 1 diabetes individuals** | | | | |
| --- | --- | --- | --- | --- |
|  | **Flash glucose monitoring system**  **N=1507 (16.8%)** | **Blood glucose monitoring system**  **N=7466 (83.2%)** | **Total**  **N=8973 (100%)** | **P value** |
| **Basal insulin prescription** | 1294 (85.9%) | 5842 (78.2%) | 7136 (79.5%) | < 0.001 |
| **Basal insulin class** |  |  |  | < 0.001 |
| **Long-acting analog** | 693 (53.6%) | 4005 (68.6%) | 4698 (65.8%) |  |
| *Detemir* | 15 (2.2%) | 70 (1.7%) | 85 (1.8%) |  |
| *Glargine* | 678 (97.8%) | 3935 (98.3%) | 4613 (98.2%) |  |
| **Long-acting analog + GLP1** | 0 (0.0%) | 0 (0.0%) | 0 (0.0%) |  |
| *Glargine + Lixisenatida* | 0 (0.0%) | 0 (0.0%) | 0 (0.0%) |  |
| **Ultra-long acting analog** | 559 (43.2%) | 851 (14.6%) | 1410 (19.8%) |  |
| *Degludec* | 533 (95.3%) | 790 (92.8%) | 1323 (93.8%) |  |
| *Glargine U300* | 26 (4.7%) | 61 (7.2%) | 87 (6.2%) |  |
| **Ultra-long acting analog + GLP1** | 4 (0.3%) | 14 (0.2%) | 18 (0.3%) |  |
| *Degludec + Liraglutida* | 4 (100%) | 14 (100%) | 18 (100%) |  |
| **Human NPH** | 38 (2.9%) | 964 (16.5%) | 1002 (14.0%) |  |
| **Mix** | 0 (0.0%) | 8 (0.1%) | 8 (0.1%) |  |
| *Humalog Mix 25* | 0 (0%) | 3 (37.5%) | 3 (37.5%) |  |
| *Humalog Mix 50* | 0 (0%) | 2 (25%) | 2 (25%) |  |
| *Humulin 70/30* | 0 (0%) | 2 (25%) | 2 (25%) |  |
| *NovoMix 30* | 0 (0%) | 1 (12.5%) | 1 (12.5%) |  |
| **Basal insulin – Total daily dose (U/day)** |  |  |  | < 0.001 |
| Mean (SD) | 22.08 (13.41) | 27.59 (14.47) | 26.60 (14.44) |  |
| Missing | 177 | 1335 | 1512 |  |
| **Basal insulin – Total daily dose per kilogram (U/Kg/day)** |  |  |  | < 0.001 |
| Mean (SD) | 0.37 (0.19) | 0.43 (0.21) | 0.42 (0.21) |  |
| Missing | 282 | 1562 | 1844 |  |
| **Basal insulin –Number of daily injections** |  |  |  | < 0.001 |
| Mean (SD) | 1.21 (0.83) | 1.53 (1.60) | 1.47 (1.50) |  |
| Missing | 175 | 1331 | 1506 |  |
| **Bolus insulin prescription** | 1232 (81.8%) | 5169 (69.2%) | 6401 (71.3%) | < 0.001 |
| **Bolus insulin class** |  |  |  | < 0.001 |
| **Short-acting analog** | 762 (61.9%) | 4165 (80.6%) | 4927 (77.0%) |  |
| *Aspart* | 404 (53%) | 2224 (53.4%) | 2628 (53.3%) |  |
| *Glulisine* | 140 (18.4%) | 1008 (24.2%) | 1148 (23.3%) |  |
| *Lispro* | 218 (28.6%) | 933 (22.4%) | 1151 (23.4%) |  |
| **Ultra-short acting analog** | 448 (36.4%) | 551 (10.7%) | 999 (15.6%) |  |
| *Fiasp* | 448 (100%) | 551 (100%) | 999 (100%) |  |
| **Human Regular** | 22 (1.8%) | 453 (8.8%) | 475 (7.4%) |  |
| **Bolus insulin – Total daily dose (U/day)** |  |  |  | < 0.001 |
| Mean (SD) | 19.57 (18.31) | 23.12 (22.28) | 22.43 (21.61) |  |
| Missing | 917 | 5012 | 5929 |  |
| **Bolus insulin – Total daily dose per kilogram (U/Kg/day)** |  |  |  | 0.505 |
| Mean (SD) | 0.34 (0.31) | 0.36 (0.35) | 0.34 (0.31) |  |
| Missing | 971 | 5097 | 6068 |  |
| **Bolus insulin – Correction factor 24hrs** |  |  |  | < 0.001 |
| Mean (SD) | 52.72 (30.68) | 45.27 (26.85) | 46.65 (27.75) |  |
| Missing | 971 | 5102 | 6073 |  |
| **Bolus insulin – Carbohydrate/insulin ratio** |  |  |  | 0.077 |
| Mean (SD) | 13.97 (9.84) | 14.02 (14.23) | 14.01 (13.52) |  |
| Missing | 971 | 5105 | 6076 |  |
| **TBD/TDD (%)** |  |  |  | 0.041 |
| Mean (SD) | 55 (17) | 57 (18) | 56 (17) |  |
| Missing | 938 | 5098 | 6036 |  |
| **Oral anti-diabetics prescription** | 73 (4.8%) | 492 (6.6%) | 565 (6.3%) | 0.011 |
| **Oral anti-diabetics class** |  |  |  | 0.036 |
| *Metformin* | 33 (45.2%) | 311 (63.2%) | 344 (60.9%) |  |
| *Sulfonylureas* | 0 (0.0%) | 14 (2.8%) | 14 (2.5%) |  |
| *Glitazones* | 1 (1.4%) | 0 (0.0%) | 1 (0.2%) |  |
| *DPP4 inhibitors* | 4 (5.5%) | 12 (2.4%) | 16 (2.8%) |  |
| *GLP1 agonists* | 4 (5.5%) | 15 (3.0%) | 19 (3.4%) |  |
| *SGLT2 inhibitors* | 17 (23.3%) | 51 (10.4%) | 68 (12.0%) |  |
| *Acarbose* | 0 (0.0%) | 6 (1.2%) | 6 (1.1%) |  |
| *Metformin + Sulfonylurea* | 2 (2.7%) | 9 (1.8%) | 11 (1.9%) |  |
| *Metformin + SGLT2 inhibitors* | 9 (12.3%) | 31 (6.3%) | 40 (7.1%) |  |
| *Metformin + DPP4 inhibitors* | 0 (0.0%) | 17 (3.5%) | 17 (3.0%) |  |
| *Other combination drugs* | 3 (4.1%) | 26 (5.3%) | 29 (5.1%) |  |

| **Supplementary Table 6 – Prescription patterns in type 2 diabetes individuals** | | | | |
| --- | --- | --- | --- | --- |
|  | **Flash glucose monitoring system**  **N=85 (3.2%)** | **Blood glucose monitoring system**  **N=2573 (96.8%)** | **Total**  **N=2658 (100%)** | **P value** |
| **Basal insulin prescription** | 43 (50.6%) | 563 (21.9%) | 606 (22.8%) | < 0.001 |
| **Basal insulin class** |  |  |  | < 0.001 |
| **Long-acting analog** | 21 (48.8%) | 149 (26.5%) | 170 (28.1%) |  |
| *Detemir* | 2 (9.5%) | 4 (2.7%) | 6 (3.5%) |  |
| *Glargine* | 19 (90.5%) | 145 (97.3%) | 164 (96.5%) |  |
| **Long-acting analog + GLP1** | 0 (0.0%) | 1 (0.2%) | 1 (0.2%) |  |
| *Glargine + Lixisenatida* | 0 (0%) | 1 (100%) | 1 (100%) |  |
| **Ultra-long acting analog** | 17 (39.5%) | 40 (7.1%) | 57 (9.4%) |  |
| *Degludec* | 16 (94.1%) | 37 (92.5%) | 53 (93%) |  |
| *Glargine U300* | 1 (5.9%) | 3 (7.5%) | 4 (7%) |  |
| **Ultra-long acting analog + GLP1** | 1 (2.3%) | 18 (3.2%) | 19 (3.1%) |  |
| *Degludec + Liraglutida* | 1 (100%) | 18 (100%) | 19 (100%) |  |
| **Human NPH** | 3 (7.0%) | 353 (62.7%) | 356 (58.7%) |  |
| **Mix** | 1 (2.3%) | 2 (0.4%) | 3 (0.5%) |  |
| *Humalog Mix 25* | 1 (100%) | 0 (0%) | 1 (33.3%) |  |
| *Humalog Mix 50* | 0 (0.0%) | 0 (0.0%) | 0 (0.0%) |  |
| *Humulin 70/30* | 0 (0%) | 2 (100%) | 2 (66.7%) |  |
| *NovoMix 30* | 0 (0.0%) | 0 (0.0%) | 0 (0.0%) |  |
| **Basal insulin – Total daily dose (U/day)** |  |  |  | 0.524 |
| Mean (SD) | 28.54 (23.15) | 29.98 (21.99) | 29.87 (22.06) |  |
| Missing | 39 | 1980 | 2019 |  |
| **Basal insulin – Total daily dose per kilogram (U/Kg/day)** |  |  |  | 0.622 |
| Mean (SD) | 0.38 (0.29) | 0.36 (0.25) | 0.36 (0.26) |  |
| Missing | 39 | 1996 | 2035 |  |
| **Basal insulin –Number of daily injections** |  |  |  | < 0.001 |
| Mean (SD) | 1.17 (0.44) | 1.62 (0.80) | 1.59 (0.79) |  |
| Missing | 39 | 1978 | 2017 |  |
| **Bolus insulin prescription** | 38 (44.7%) | 231 (9.0%) | 269 (10.1%) | < 0.001 |
| **Bolus insulin class** |  |  |  | < 0.001 |
| **Short-acting analog** | 15 (39.5%) | 81 (35.1%) | 96 (35.7%) |  |
| *Aspart* | 5 (33.3%) | 31 (38.3%) | 36 (37.5%) |  |
| *Glulisine* | 6 (40%) | 31 (38.3%) | 37 (38.5%) |  |
| *Lispro* | 4 (26.7%) | 19 (23.5%) | 23 (24%) |  |
| **Ultra-short acting analog** | 21 (55.3%) | 30 (13.0%) | 51 (19.0%) |  |
| *Fiasp* | 21 (100%) | 30 (100%) | 51 (100%) |  |
| **Human Regular** | 2 (5.3%) | 120 (51.9%) | 122 (45.4%) |  |
| **Bolus insulin – Total daily dose (U/day)** |  |  |  | 0.651 |
| Mean (SD) | 26.83 (22.13) | 31.01 (37.35) | 30.27 (35.07) |  |
| Missing | 67 | 2490 | 2557 |  |
| **Bolus insulin – Total daily dose per kilogram (U/Kg/day)** |  |  |  | 0.267 |
| Mean (SD) | 0.36 (0.29) | 0.37 (0.50) | 0.37 (0.47) |  |
| Missing | 67 | 2494 | 2561 |  |
| **Bolus insulin – Correction factor 24hrs** |  |  |  | 0.516 |
| Mean (SD) | 33.89 (16.76) | 37.03 (35.11) | 36.44 (32.42) |  |
| Missing | 67 | 2495 | 2562 |  |
| **Bolus insulin – Carbohydrate/insulin ratio** |  |  |  | 0.879 |
| Mean (SD) | 11.89 (4.56) | 30.16 (94.41) | 26.69 (85.21) |  |
| Missing | 67 | 2496 | 2563 |  |
| **TBD/TDD (%)** |  |  |  | 0.141 |
| Mean (SD) | 50 (21) | 58 (24) | 56 (23) |  |
| Missing | 69 | 2497 | 2566 |  |
| **Oral anti-diabetics prescription** | 20 (23.5%) | 890 (34.6%) | 910 (34.2%) | 0.034 |
| **Oral anti-diabetics class** |  |  |  | < 0.001 |
| *Metformin* | 7 (35.0%) | 395 (44.4%) | 402 (44.2%) |  |
| *Sulfonylureas* | 0 (0.0%) | 47 (5.3%) | 47 (5.2%) |  |
| *Glitazones* | 0 (0.0%) | 6 (0.7%) | 6 (0.7%) |  |
| *DPP4 inhibitors* | 1 (5.0%) | 11 (1.2%) | 12 (1.3%) |  |
| *GLP1 agonists* | 0 (0.0%) | 4 (0.4%) | 4 (0.4%) |  |
| *SGLT2 inhibitors* | 2 (10.0%) | 26 (2.9%) | 28 (3.1%) |  |
| *Acarbose* | 0 (0.0%) | 0 (0.0%) | 0 (0.0%) |  |
| *Metformin + Sulfonylurea* | 0 (0.0%) | 131 (14.7%) | 131 (14.4%) |  |
| *Metformin + SGLT2 inhibitors* | 3 (15.0%) | 94 (10.6%) | 97 (10.7%) |  |
| *Metformin + DPP4 inhibitors* | 1 (5.0%) | 37 (4.2%) | 38 (4.2%) |  |
| *Other combination drugs* | 6 (30.0%) | 139 (15.6%) | 145 (15.9%) |  |

| **Supplementary Table 7 – Prescription patterns in gestational diabetes individuals** | | | | |
| --- | --- | --- | --- | --- |
|  | **Flash glucose monitoring system**  **N=11 (1.9%)** | **Blood glucose monitoring system**  **N=562 (98.1%)** | **Total**  **N=573 (100%)** | **P value** |
| **Basal insulin prescription** | 3 (27.3%) | 39 (6.9%) | 42 (7.3%) | 0.010 |
| **Basal insulin class** |  |  |  | < 0.001 |
| **Long-acting analog** | 2 (66.7%) | 3 (7.7%) | 5 (11.9%) |  |
| *Detemir* | 0 (0%) | 3 (100%) | 3 (60%) |  |
| *Glargine* | 2 (100%) | 0 (0%) | 2 (40%) |  |
| **Long-acting analog + GLP1** | 0 (0.0%) | 0 (0.0%) | 0 (0.0%) |  |
| *Glargine + Lixisenatida* | 0 (0.0%) | 0 (0.0%) | 0 (0.0%) |  |
| **Ultra-long acting analog** | 1 (33.3%) | 1 (2.6%) | 2 (4.8%) |  |
| *Degludec* | 1 (100%) | 1 (100%) | 2 (100%) |  |
| *Glargine U300* | 0 (0.0%) | 0 (0.0%) | 0 (0.0%) |  |
| **Ultra-long acting analog + GLP1** | 0 (0.0%) | 0 (0.0%) | 0 (0.0%) |  |
| *Degludec + Liraglutida* | 0 (0.0%) | 0 (0.0%) | 0 (0.0%) |  |
| **Human NPH** | 0 (0.0%) | 35 (89.7%) | 35 (83.3%) |  |
| **Mix** | 0 (0.0%) | 0 (0.0%) | 0 (0.0%) |  |
| *Humalog Mix 25* | 0 (0.0%) | 0 (0.0%) | 0 (0.0%) |  |
| *Humalog Mix 50* | 0 (0.0%) | 0 (0.0%) | 0 (0.0%) |  |
| *Humulin 70/30* | 0 (0.0%) | 0 (0.0%) | 0 (0.0%) |  |
| *NovoMix 30* | 0 (0.0%) | 0 (0.0%) | 0 (0.0%) |  |
| **Basal insulin – Total daily dose (U/day)** |  |  |  | 0.371 |
| Mean (SD) | 26.67 (20.43) | 20.84 (20.84) | 21.21 (20.64) |  |
| Missing | 8 | 518 | 526 |  |
| **Basal insulin – Total daily dose per kilogram (U/Kg/day)** |  |  |  | 0.161 |
| Mean (SD) | 0.33 (0.14) | 0.24 (0.24) | 0.25 (0.24) |  |
| Missing | 8 | 519 | 527 |  |
| **Basal insulin –Number of daily injections** |  |  |  | 0.595 |
| Mean (SD) | 1.33 (0.58) | 1.70 (0.93) | 1.68 (0.91) |  |
| Missing | 8 | 518 | 526 |  |
| **Bolus insulin prescription** | 4 (36.4%) | 11 (2.0%) | 15 (2.6%) | < 0.001 |
| **Bolus insulin class** |  |  |  | < 0.001 |
| **Short-acting analog** | 4 (100.0%) | 0 (0.0%) | 4 (26.7%) |  |
| *Aspart* | 3 (75%) | 0 (0%) | 3 (75%) |  |
| *Glulisine* | 0 (0.0%) | 0 (0.0%) | 0 (0.0%) |  |
| *Lispro* | 1 (25%) | 0 (0%) | 1 (25%) |  |
| **Ultra-short acting analog** | 0 (0.0%) | 2 (18.2%) | 2 (13.3%) |  |
| *Fiasp* | 0 (0%) | 2 (100%) | 2 (100%) |  |
| **Human Regular** | 0 (0.0%) | 9 (81.8%) | 9 (60.0%) |  |
| **Bolus insulin – Total daily dose (U/day)** |  |  |  | 0.221 |
| Mean (SD) | 20.50 (12.02) | 3.00 (NA) | 14.67 (13.20) |  |
| Missing | 9 | 561 | 570 |  |
| **Bolus insulin – Total daily dose per kilogram (U/Kg/day)** |  |  |  | 0.221 |
| Mean (SD) | 0.23 (0.07) | 0.04 (NA) | 0.17 (0.12) |  |
| Missing | 9 | 561 | 570 |  |
| **Bolus insulin – Correction factor 24hrs** |  |  |  | 0.480 |
| Mean (SD) | 42.50 (3.54) | 40.00 (NA) | 41.67 (2.89) |  |
| Missing | 9 | 561 | 570 |  |
| **Bolus insulin – Carbohydrate/insulin ratio** |  |  |  | 0.221 |
| Mean (SD) | 9.00 (4.24) | 1.00 (NA) | 6.33 (5.51) |  |
| Missing | 9 | 561 | 570 |  |
| **TBD/TDD (%)** |  |  |  | 0.221 |
| Mean (SD) | 62 (2) | 96 (NA) | 73 (20) |  |
| Missing | 9 | 561 | 570 |  |
| **Oral anti-diabetics prescription** | 2 (18.2%) | 14 (2.5%) | 16 (2.8%) | 0.002 |
| **Oral anti-diabetics class** |  |  |  | 0.003 |
| *Metformin* | 2 (100.0%) | 14 (100.0%) | 16 (100.0%) |  |
| *Sulfonylureas* | 0 (0.0%) | 0 (0.0%) | 0 (0.0%) |  |
| *Glitazones* | 0 (0.0%) | 0 (0.0%) | 0 (0.0%) |  |
| *DPP4 inhibitors* | 0 (0.0%) | 0 (0.0%) | 0 (0.0%) |  |
| *GLP1 agonists* | 0 (0.0%) | 0 (0.0%) | 0 (0.0%) |  |
| *SGLT2 inhibitors* | 0 (0.0%) | 0 (0.0%) | 0 (0.0%) |  |
| *Acarbose* | 0 (0.0%) | 0 (0.0%) | 0 (0.0%) |  |
| *Metformin + Sulfonylurea* | 0 (0.0%) | 0 (0.0%) | 0 (0.0%) |  |
| *Metformin + SGLT2 inhibitors* | 0 (0.0%) | 0 (0.0%) | 0 (0.0%) |  |
| *Metformin + DPP4 inhibitors* | 0 (0.0%) | 0 (0.0%) | 0 (0.0%) |  |
| *Other combination drugs* | 0 (0.0%) | 0 (0.0%) | 0 (0.0%) |  |

| **Supplementary Table 8 – Prescription patterns in LADA individuals** | | | | |
| --- | --- | --- | --- | --- |
|  | **Flash glucose monitoring system**  **N=117 (22.4%)** | **Blood glucose monitoring system**  **N=406 (77.6%)** | **Total**  **N=523 (100%)** | **P value** |
| **Basal insulin prescription** | 101 (86.3%) | 362 (89.2%) | 463 (88.5%) | 0.396 |
| **Basal insulin class** |  |  |  | < 0.001 |
| **Long-acting analog** | 49 (48.5%) | 209 (57.7%) | 258 (55.7%) |  |
| *Detemir* | 1 (2%) | 4 (1.9%) | 5 (1.9%) |  |
| *Glargine* | 48 (98%) | 205 (98.1%) | 253 (98.1%) |  |
| **Long-acting analog + GLP1** | 0 (0.0%) | 0 (0.0%) | 0 (0.0%) |  |
| *Glargine + Lixisenatida* | 0 (0.0%) | 0 (0.0%) | 0 (0.0%) |  |
| **Ultra-long acting analog** | 50 (49.5%) | 75 (20.7%) | 125 (27.0%) |  |
| *Degludec* | 49 (98%) | 69 (92%) | 118 (94.4%) |  |
| *Glargine U300* | 1 (2%) | 6 (8%) | 7 (5.6%) |  |
| **Ultra-long acting analog + GLP1** | 2 (2.0%) | 3 (0.8%) | 5 (1.1%) |  |
| *Degludec + Liraglutida* | 2 (100%) | 3 (100%) | 5 (100%) |  |
| **Human NPH** | 0 (0.0%) | 75 (20.7%) | 75 (16.2%) |  |
| **Mix** | 0 (0.0%) | 0 (0.0%) | 0 (0.0%) |  |
| *Humalog Mix 25* | 0 (0.0%) | 0 (0.0%) | 0 (0.0%) |  |
| *Humalog Mix 50* | 0 (0.0%) | 0 (0.0%) | 0 (0.0%) |  |
| *Humulin 70/30* | 0 (0.0%) | 0 (0.0%) | 0 (0.0%) |  |
| *NovoMix 30* | 0 (0.0%) | 0 (0.0%) | 0 (0.0%) |  |
| **Basal insulin – Total daily dose (U/day)** |  |  |  | < 0.001 |
| Mean (SD) | 19.99 (11.40) | 26.47 (17.87) | 25.07 (16.89) |  |
| Missing | 14 | 33 | 47 |  |
| **Basal insulin – Total daily dose per kilogram (U/Kg/day)** |  |  |  | < 0.001 |
| Mean (SD) | 0.28 (0.13) | 0.37 (0.23) | 0.35 (0.22) |  |
| Missing | 15 | 34 | 49 |  |
| **Basal insulin –Number of daily injections** |  |  |  | < 0.001 |
| Mean (SD) | 1.14 (0.40) | 1.45 (0.78) | 1.38 (0.72) |  |
| Missing | 14 | 31 | 45 |  |
| **Bolus insulin prescription** | 89 (76.1%) | 288 (70.9%) | 377 (72.1%) | 0.276 |
| **Bolus insulin class** |  |  |  | < 0.001 |
| **Short-acting analog** | 50 (56.2%) | 200 (69.4%) | 250 (66.3%) |  |
| *Aspart* | 27 (54%) | 102 (51%) | 129 (51.6%) |  |
| *Glulisine* | 13 (26%) | 61 (30.5%) | 74 (29.6%) |  |
| *Lispro* | 10 (20%) | 37 (18.5%) | 47 (18.8%) |  |
| **Ultra-short acting analog** | 38 (42.7%) | 46 (16.0%) | 84 (22.3%) |  |
| *Fiasp* | 38 (100%) | 46 (100%) | 84 (100%) |  |
| **Human Regular** | 1 (1.1%) | 42 (14.6%) | 43 (11.4%) |  |
| **Bolus insulin – Total daily dose (U/day)** |  |  |  | 0.772 |
| Mean (SD) | 22.02 (21.27) | 24.92 (30.52) | 24.27 (28.69) |  |
| Missing | 75 | 260 | 335 |  |
| **Bolus insulin – Total daily dose per kilogram (U/Kg/day)** |  |  |  | 0.686 |
| Mean (SD) | 0.30 (0.28) | 0.36 (0.46) | 0.34 (0.43) |  |
| Missing | 76 | 261 | 337 |  |
| **Bolus insulin – Correction factor 24hrs** |  |  |  | 0.762 |
| Mean (SD) | 44.27 (19.31) | 75.14 (215.71) | 68.33 (190.96) |  |
| Missing | 76 | 261 | 337 |  |
| **Bolus insulin – Carbohydrate/insulin ratio** |  |  |  | 0.744 |
| Mean (SD) | 14.24 (8.10) | 14.57 (12.47) | 14.50 (11.62) |  |
| Missing | 76 | 262 | 338 |  |
| **TBD/TDD (%)** |  |  |  | 0.369 |
| Mean (SD) | 55 (18) | 58 (20) | 57 (20) |  |
| Missing | 78 | 261 | 339 |  |
| **Oral anti-diabetics prescription** | 15 (12.8%) | 68 (16.7%) | 83 (15.9%) | 0.306 |
| **Oral anti-diabetics class** |  |  |  | 0.242 |
| *Metformin* | 4 (26.7%) | 40 (58.8%) | 44 (53.0%) |  |
| *Sulfonylureas* | 0 (0.0%) | 1 (1.5%) | 1 (1.2%) |  |
| *Glitazones* | 0 (0.0%) | 1 (1.5%) | 1 (1.2%) |  |
| *DPP4 inhibitors* | 2 (13.3%) | 3 (4.4%) | 5 (6.0%) |  |
| *GLP1 agonists* | 0 (0.0%) | 0 (0.0%) | 0 (0.0%) |  |
| *SGLT2 inhibitors* | 1 (6.7%) | 4 (5.9%) | 5 (6.0%) |  |
| *Acarbose* | 1 (6.7%) | 0 (0.0%) | 1 (1.2%) |  |
| *Metformin + Sulfonylurea* | 0 (0.0%) | 1 (1.5%) | 1 (1.2%) |  |
| *Metformin + SGLT2 inhibitors* | 3 (20.0%) | 6 (8.8%) | 9 (10.8%) |  |
| *Metformin + DPP4 inhibitors* | 2 (13.3%) | 5 (7.4%) | 7 (8.4%) |  |
| *Other combination drugs* | 2 (13.3%) | 7 (10.3%) | 9 (10.8%) |  |

| **Supplementary Table 9. Population characteristics of type 1 diabetes and LADA individuals who attended the glycemic control analyses requirements** | | | | |
| --- | --- | --- | --- | --- |
|  | **Flash glucose monitoring system**  **N=120 (17.2%)** | **Blood glucose monitoring system**  **N=578 (82.8%)** | **Total**  **N=698 (100%)** | **P value** |
| **Type of diabetes** |  |  |  | 0.092 |
| Type 1 | 109 (90.8%) | 548 (94.8%) | 657 (94.1%) |  |
| LADA | 11 (9.2%) | 30 (5.2%) | 41 (5.9%) |  |
| **Age** |  |  |  | 0.176 |
| Mean (SD) | 31.33 (20.99) | 32.53 (15.35) | 32.32 (16.45) |  |
| **Sex** |  |  |  | 0.811 |
| Female | 63 (53.4%) | 303 (54.6%) | 366 (54.4%) |  |
| Male | 55 (46.6%) | 252 (45.4%) | 307 (45.6%) |  |
| Missing | 2 | 23 | 25 |  |
| **Region** |  |  |  | 0.024 |
| Central-west | 8 (8.4%) | 28 (5.8%) | 36 (6.2%) |  |
| Northeast | 5 (5.3%) | 57 (11.7%) | 62 (10.7%) |  |
| North | 3 (3.2%) | 2 (0.4%) | 5 (0.9%) |  |
| Southeast | 56 (58.9%) | 293 (60.3%) | 349 (60.1%) |  |
| South | 23 (24.2%) | 106 (21.8%) | 129 (22.2%) |  |
| Missing | 25 | 92 | 117 |  |
| **Years of diabetes diagnosis** |  |  |  | 0.157 |
| Mean (SD) | 10.68 (10.37) | 12.45 (11.33) | 12.12 (11.17) |  |
| Missing | 12 | 100 | 112 |  |
| **BMI (kg/m^2^)** |  |  |  | 0.092 |
| Mean (SD) | 23.28 (5.26) | 24.05 (4.32) | 23.93 (4.49) |  |
| Missing | 19 | 46 | 65 |  |
| **Frequency of Glic use** |  |  |  | 0.276 |
| Mean (SD) | 114.52 (184.71) | 93.93 (80.59) | 97.47 (106.12) |  |

| **Supplementary Table 10. Comparison of population characteristics of all eligible individuals and those type 1 diabetes and LADA individuals who attended the glycemic control analyses requirements** | | | | |
| --- | --- | --- | --- | --- |
|  | **All eligible individuals**  **N=12029 (94.5%)** | **Glycemic control cohort**  **N=698 (5.5%)** | **Total**  **N=12727 (100%)** | **P value** |
| **Age** |  |  |  | < 0.001 |
| Mean (SD) | 35.80 (16.11) | 32.32 (16.45) | 35.61 (16.14) |  |
| **Sex** |  |  |  | < 0.001 |
| Female | 7127 (61.3%) | 366 (54.4%) | 7493 (60.9%) |  |
| Male | 4496 (38.7%) | 307 (45.6%) | 4803 (39.1%) |  |
| Missing | 406 | 25 | 431 |  |
| **Region** |  |  |  | < 0.001 |
| Central-west | 938 (9.3%) | 36 (6.2%) | 974 (9.1%) |  |
| Northeast | 1405 (13.9%) | 62 (10.7%) | 1467 (13.7%) |  |
| North | 266 (2.6%) | 5 (0.9%) | 271 (2.5%) |  |
| Southeast | 5750 (56.9%) | 349 (60.1%) | 6099 (57.0%) |  |
| South | 1755 (17.4%) | 129 (22.2%) | 1884 (17.6%) |  |
| Missing | 1915 | 117 | 2032 |  |
| **Years of diabetes diagnosis** |  |  |  | < 0.001 |
| Mean (SD) | 8.94 (10.35) | 12.12 (11.17) | 9.10 (10.41) |  |
| Missing | 1190 | 112 | 1302 |  |
| **BMI (kg/m^2^)** |  |  |  | < 0.001 |
| Mean (SD) | 25.75 (5.36) | 23.93 (4.49) | 25.66 (5.34) |  |
| Missing | 880 | 65 | 945 |  |
| **Frequency of Glic use** |  |  |  | < 0.001 |
| Mean (SD) | 26.41 (112.86) | 198.00 (198.96) | 35.82 (125.43) |  |

| **Table 11 – Glucose levels of the type 1 diabetes and LADA in adults (>18 years)** | | | | |
| --- | --- | --- | --- | --- |
|  | **Flash glucose monitoring system**  **(N=79)** | **Blood glucose monitoring system**  **(N=493)** | **Total**  **(N=572)** | **P value** |
| **24hrs glucose level (mg/dL)** |  |  |  | 0.467 |
| Mean (SD) | 174.27 (38.90) | 179.80 (48.55) | 179.04 (47.34) |  |
| Missing | 0 | 0 | 0 |  |
| **Nocturnal glucose level (mg/dL)** |  |  |  | 0.013 |
| Mean (SD) | 223.24 (74.82) | 188.98 (89.11) | 193.32 (88.04) |  |
| Missing | 46 | 266 | 312 |  |
| **Pre-prandial glucose level (mg/dL)** |  |  |  | 0.127 |
| Mean (SD) | 165.33 (33.36) | 175.14 (47.04) | 173.78 (45.49) |  |
| Missing | 2 | 16 | 18 |  |
| **Post-prandial glucose level (mg/dL)** |  |  |  | 0.652 |
| Mean (SD) | 188.74 (62.36) | 185.97 (64.52) | 186.36 (64.16) |  |
| Missing | 3 | 38 | 41 |  |
| **Glycemic control categories** |  |  |  |  |
| *Normoglycemia* | 55% | 52% | 52% |  |
| *Level 2 hypoglycemia* | 1% | 2% | 2% |  |
| *Level 1 hypoglycemia* | 3% | 4% | 4% |  |
| *Level 1 hyperglycemia* | 24% | 23% | 23% |  |
| *Level 2 hyperglycemia* | 16% | 20% | 19% |  |

| **Table 12 – Glucose levels of the type 1 diabetes and LADA in children and adolescents (<18 years)** | | | | |
| --- | --- | --- | --- | --- |
|  | **Flash glucose monitoring system**  **(N=41)** | **Blood glucose monitoring system**  **(N=85)** | **Total**  **(N=126)** | **P value** |
| **24hrs glucose level (mg/dL)** |  |  |  | 0.072 |
| Mean (SD) | 175.18 (40.83) | 190.75 (44.52) | 185.69 (43.81) |  |
| Missing | 0 | 0 | 0 |  |
| **Nocturnal glucose level (mg/dL)** |  |  |  | 0.330 |
| Mean (SD) | 237.38 (80.35) | 211.25 (70.51) | 215.52 (71.97) |  |
| Missing | 33 | 44 | 77 |  |
| **Pre-prandial glucose level (mg/dL)** |  |  |  | 0.060 |
| Mean (SD) | 171.13 (39.00) | 188.02 (45.99) | 182.48 (44.39) |  |
| Missing | 0 | 1 | 1 |  |
| **Post-prandial glucose level (mg/dL)** |  |  |  | 0.085 |
| Mean (SD) | 173.11 (49.68) | 193.26 (65.60) | 186.71 (61.41) |  |
| Missing | 2 | 4 | 6 |  |
| **Glycemic control categories** |  |  |  |  |
| *Normoglycemia* | 56% | 49% | 51% |  |
| *Level 2 hypoglycemia* | 1% | 1% | 1% |  |
| *Level 1 hypoglycemia* | 3% | 3% | 3% |  |
| *Level 1 hyperglycemia* | 22% | 23% | 23% |  |
| *Level 2 hyperglycemia* | 18% | 24% | 22% |  |
